# Supplementary material for: The Systems Biology Research Tool: evolvable open-source software
Source: BMC Syst Biol. 2008 Jun 29;2:55. doi: 10.1186/1752-0509-2-55 (PMC2446383; doi:10.1186/1752-0509-2-55)
Supplement: Additional file 1 — SBRT Archive. An archive of the current version of the Systems Biology Research Tool. [file 1752-0509-2-55-S1.zip › sbrt-1.4.0/doc/developers_guide/api/sbrt/kernel/fba/comp/class-use/FbaOptComputer.Results.html]

Uses of Class sbrt.kernel.fba.comp.FbaOptComputer.Results


|  |  |  |
| --- | --- | --- |
| **Overview** \| **Package** \| **Class** \| **Use** \| **Tree** \| **Deprecated** \| **Index** \| **Help** | |  |
| PREV   NEXT | **FRAMES**    **NO FRAMES**     **All Classes** |  |


---


## **Uses of Class sbrt.kernel.fba.comp.FbaOptComputer.Results**

| Packages that use FbaOptComputer.Results | |
| --- | --- |
| **sbrt.kernel.fba.comp** | Provides classes and interfaces for performing Flux Balance Analysis computations. |
| **sbrt.kernel.fba.proc** | Provides classes and interfaces for executing Flux Balance Analysis processes. |
| **sbrt.shell.io.fba** | Provides classes and interfaces for reading and writing files relevant to Flux Balance Analysis. |
| **sbrt.shell.mng.fba** | Provides classes and interfaces for creating Flux Balance Analysis process managers. |

Uses of FbaOptComputer.Results in sbrt.kernel.fba.comp

| Methods in sbrt.kernel.fba.comp that return FbaOptComputer.Results | |
| --- | --- |
| `FbaOptComputer.Results` | `RxnDeletionComputer.delete(java.util.Set<java.lang.String> rxnNames)`             Computes and returns the results of deleting the reactions whose names appear in the provided set. |
| `FbaOptComputer.Results` | `FbaOptComputer.getMax(MathExpr<?> objective)`             Returns the results of computing the maximum value of the provided objective function. |
| `FbaOptComputer.Results` | `FbaOptComputer.getMin(MathExpr<?> objective)`             Returns the results of computing the minimum value of the provided objective function. |
| `FbaOptComputer.Results` | `FbaOptComputer.getOptimal(MathExpr<?> objective, OptimizationSense sense)`             Returns the results of computing the optimal value of the provided objective function. |
| `FbaOptComputer.Results` | `FbaOptComputer.getOptimal(java.lang.String reactionName, OptimizationSense sense)`             Returns the results of computing the optimal flux of the reaction with the provided name. |

Uses of FbaOptComputer.Results in sbrt.kernel.fba.proc

| Method parameters in sbrt.kernel.fba.proc with type arguments of type FbaOptComputer.Results | |
| --- | --- |
| `void` | `CvFbaOptimizationExperiment.run(ResultsManager<FbaOptComputer.Results> resultsManager)`             Runs this process and adds the generated results to the provided results manager via its `ResultsManager.addData(Object)` method. |
| `void` | `ObjectiveFunctionAnalysis.run(ResultsManager<FbaOptComputer.Results> resultsManager)`             Runs this process and adds the generated results to the provided results manager via its `ResultsManager.addData(Object)` method. |
| `void` | `CatalystDeletionProcess.run(ResultsManager<FbaOptComputer.Results> resultsManager)`             Runs this process and adds the generated results to the provided results manager via its `ResultsManager.addData(Object)` method. |
| `void` | `RxnDeletionProcess.run(ResultsManager<FbaOptComputer.Results> resultsManager)`             Runs this process and adds the generated results to the provided results manager via its `ResultsManager.addData(Object)` method. |
| `void` | `CvRxnDeletionProcess.run(ResultsManager<FbaOptComputer.Results> resultsManager)`             Runs this process and adds the generated results to the provided results manager via its `ResultsManager.addData(Object)` method. |
| `void` | `StoichVariationProcess.run(ResultsManager<FbaOptComputer.Results> resultsManager)`             Runs this process and adds the generated results to the provided results manager via its `ResultsManager.addData(Object)` method. |
| `void` | `CvObjectiveFunctionProcess.run(ResultsManager<FbaOptComputer.Results> resultsManager)`             Runs this process and adds the generated results to the provided results manager via its `ResultsManager.addData(Object)` method. |
| `void` | `FbaOptimizationProcess.run(ResultsManager<FbaOptComputer.Results> resultsManager)`             Computes the optimal value of the current objective function and adds the results to the provided results manager via its `ResultsManager.addData(Object)` method. |
| `void` | `CvCatalystDeletionProcess.run(ResultsManager<FbaOptComputer.Results> resultsManager)`             Runs this process and adds the generated results to the provided results manager via its `ResultsManager.addData(Object)` method. |

Uses of FbaOptComputer.Results in sbrt.shell.io.fba

| Methods in sbrt.shell.io.fba with type parameters of type FbaOptComputer.Results | |
| --- | --- |
| `<S extends FbaOptComputer.Results> void` | `ColumnOrFbaOptOutputFile.addData(S results)`             Writes the appropriate values in the provided `FbaOptimizationComputer.Results` to this file. |
| `<S extends FbaOptComputer.Results> void` | `SerFbaOptOutputFile.addData(S results)`             Writes the appropriate values in the provided `FbaOptimizationComputer.Results` to this file. |
| `<S extends FbaOptComputer.Results> void` | `RowOrFbaOptOutputFile.addData(S results)`             Writes the appropriate values in the provided `FbaOptimizationComputer.Results` to this file. |

Uses of FbaOptComputer.Results in sbrt.shell.mng.fba

| Methods in sbrt.shell.mng.fba that return types with arguments of type FbaOptComputer.Results | |
| --- | --- |
| `static MultiOutputFiles<ColumnOrFbaOptOutputFile,FbaOptComputer.Results>` | `FbaOptManager.getMultiColOrOutputFiles(ManagerInput<java.lang.String,java.lang.String> input, java.io.PrintWriter out, java.util.Iterator<java.lang.String> fileNameSource, java.lang.String fileNameSourceName, Fluxome fluxome)`             Please consult the source code for a complete description of this method's actions. |
| `static MultiOutputFiles<? extends FbaOptOutputFile,FbaOptComputer.Results>` | `FbaOptManager.getMultiFileOutput(ManagerInput<java.lang.String,java.lang.String> input, java.io.PrintWriter out, java.util.Iterator<java.lang.String> fileNameSource, java.lang.String fileNameSourceName, Fluxome fluxome)`             Please consult the source code for a complete description of this method's actions. |
| `static MultiOutputFiles<RowOrFbaOptOutputFile,FbaOptComputer.Results>` | `FbaOptManager.getMultiRowOrOutputFiles(ManagerInput<java.lang.String,java.lang.String> input, java.io.PrintWriter out, java.util.Iterator<java.lang.String> fileNameSource, Fluxome fluxome)`             Please consult the source code for a complete description of this method's actions. |
| `static MultiOutputFiles<SerFbaOptOutputFile,FbaOptComputer.Results>` | `FbaOptManager.getMultiSerOutputFiles(ManagerInput<java.lang.String,java.lang.String> input, java.io.PrintWriter out, java.util.Iterator<java.lang.String> fileNameSource, java.lang.String fileNameSourceName, Fluxome fluxome)`             Please consult the source code for a complete description of this method's actions. |

---


|  |  |  |
| --- | --- | --- |
| **Overview** \| **Package** \| **Class** \| **Use** \| **Tree** \| **Deprecated** \| **Index** \| **Help** | |  |
| PREV   NEXT | **FRAMES**    **NO FRAMES**     **All Classes** |  |


---
